# Combined Effects of Texture and Grain Size Distribution on the Tensile Behavior of *α*-Titanium

**DOI:** 10.3390/ma11071088

**Published:** 2018-06-26

**Authors:** Thiebaud Richeton, Francis Wagner, Cai Chen, Laszlo S. Toth

**Affiliations:** 1 Université de Lorraine, CNRS, Arts et Métiers ParisTech, LEM3, F-57000 Metz, France; francis.wagner@univ-lorraine.fr (F.W.); laszlo.toth@univ-lorraine.fr (L.S.T.); 2 Laboratory of Excellence on Design of Alloy Metals for Low-Mass Structures (DAMAS), Université de Lorraine, 57073 Metz, France; cai.chen@njust.edu.cn; 3 Nanjing University of Science and Technology, 210094 Nanjing, China

**Keywords:** grain size, texture, crystal plasticity, elasto-visco-plastic self-consistent (EVPSC) scheme, hardening, dislocation density, titanium

## Abstract

This work analyzes the role of both the grain size distribution and the crystallographic texture on the tensile behavior of commercially pure titanium. Specimens with different microstructures, especially with several mean grain sizes, were specifically prepared for that purpose. It is observed that the yield stress depends on the grain size following a Hall–Petch relationship, that the stress–strain curves have a tendency to form a plateau that becomes more and more pronounced with decreasing mean grain size and that the hardening capacity increases with the grain size. All these observations are well reproduced by an elasto-visco-plastic self-consistent model that incorporates grain size effects within a crystal plasticity framework where dislocations’ densities are the state variables. First, the critical resolved shear stresses are made dependent on the individual grain size through the addition of a Hall–Petch type term. Then, the main originality of the model comes from the fact that the multiplication of mobile dislocation densities is also made grain size dependent. The underlying assumption is that grain boundaries act mainly as barriers or sinks for dislocations. Hence, the smaller the grain size, the smaller the expansion of dislocation loops and thus the smaller the increase rate of mobile dislocation density is. As a consequence of this hypothesis, both mobile and forest dislocation densities increase with the grain size and provide an explanation for the grain size dependence of the transient low work hardening rate and hardening capacity.

## 1. Introduction

Both crystallographic texture and grain size distribution are recognized to be of prime importance to understand the mechanical behavior of polycrystals [1,2,3,4]. In particular, since the works of Hall [5] and Petch [6], the grain size is known to strongly influence the yield stress of polycrystals. For grain sizes larger than 1 µm, the work hardening behavior is, however, generally reported to become almost insensitive to the grain size after a few percent of strain, particularly in face centered cubic metals [7,8,9]. Texture effects arise because of elastic and plastic anisotropies at the crystal level and are well captured by homogenization schemes like self-consistent approaches which can handle an extensive number of crystallographic orientations within reasonable CPU time simulations. However, only a few approaches do account explicitly for grain size distribution in addition to crystallographic texture [2,4,10,11,12,13]. In these mean-field approaches, the individual grain sizes get into the constitutive laws of the model as ad hoc parameters. Otherwise, no grain size effect could be predicted since these classical homogenization schemes do not include any internal length scale. Predicting direct grain size effects is, however, possible from strain gradient theories or generalized continuum plasticity models (e.g., [14]). Though not the grain size, these models consider nevertheless an internal length scale parameter whose value is generally also defined in an ad hoc way. Since the equivalent grain diameter, similarly to the grain orientation Euler angles, can now be readily determined by EBSD analyses, considering the individual grain size in self-consistent models still remains an attractive approach due to its potential good compromise between relative model simplicity, low CPU time and reliability of the predictions. The question is, however, to know how to modify the constitutive laws to include relevant effects of the individual grain size.

The present work focuses on the tensile behavior of commercially pure (cp) α-titanium which exhibits a hexagonal close-packed (hcp) crystallographic structure. In particular, α-Ti is characterized by a high anisotropy of glide resistance [15] and also by non-negligible anisotropy of slip family rate dependence [16,17,18,19,20,21,22]. Tensile tests were performed on Ti specimens which were specifically prepared to exhibit various microstructures in order to study the combined role of the texture and the grain size distribution. On the other hand, the constitutive setting of an elasto-visco-plastic self-consistent (EVPSC) scheme [19,23,24] is modified in order to include grain size effects. The constitutive framework is based on crystal plasticity and considers dislocations’ densities as state variables. The first goal of the present paper is to present an original way to account for the grain size effect in the dislocation evolution laws. Then, by comparison of the experimental and simulated results, its other purpose is to clarify the respective role of the texture and the grain size distribution and to contribute to a better understanding of the tensile hardening behavior of cp Ti.

The paper is organized as follows. Section 2.1 presents experimental details. Section 2.2 describes the micromechanical model and the way grain size effects are considered. Section 3 shows and discusses the simulations results in comparison with the experimental characterizations. Concluding remarks follow.

## 2. Materials and Methods

### 2.1. Preparation, Microstructural and Mechanical Characterization of the Specimens

**Figure 1:**
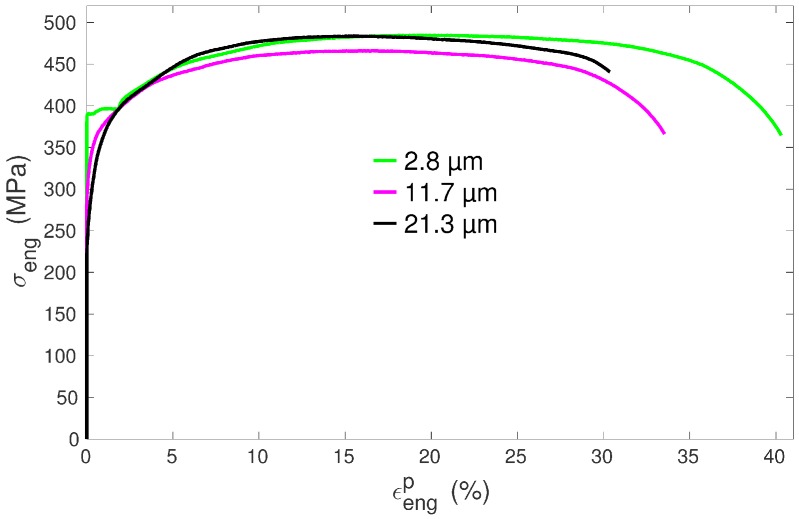
Engineering stress versus engineering plastic strain for three specimens of RR type with different mean grain sizes.

The as-received material was a 2 mm-thick plate of commercially pure titanium (Ti grade 2) in a fully recrystallized state with a mean grain size $D_{m}$ of about 10 µm. Specimens cut off from this plate were cold rolled, either along the previous rolling direction or along the previous transverse direction. After rolling, the specimens were annealed under various conditions in a furnace working under secondary vacuum. The samples were small dog-bone-shaped specimens with total length $L_{0}=43$ mm, gauge length $L_{g}=11.0$ mm, width $w_{0}=3.0$ mm, thickness $t_{0}=0.5$ mm and could be put entirely in an FEG-SEM. Tensile tests were performed at ambient temperature on a Deben Microtest MT10331-1kN tensile machine (Woolpit, UK) at a constant displacement speed of 0.5 mm·mn^−1^, which corresponds to an initial strain rate of $7.5\cdot 10^{-4}$ s^−1^. The extension direction was parallel either to the last rolling direction or to the last transverse direction. Throughout the paper, the nomenclature to name the specimens is the following: the first letter corresponds to the last rolling direction (R means rolling along the previous rolling direction and T along the previous transverse direction) whereas the second letter refers to the direction of tension (R means tension along the last rolling direction and T along the last transverse direction) and the number to the mean grain size in microns. The names of the seven selected specimens used in this study and their conditions of preparation are reported in Table 1. Figure 1 exhibits examples of experimental tensile curves obtained for three specimens of RR type (i.e., rolling and tension along the previous rolling direction) with different mean grain sizes.

**Figure 2:**
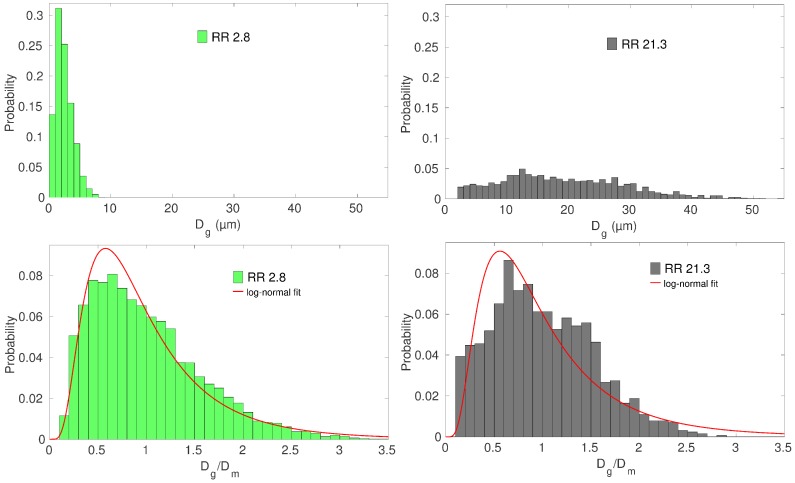
Probability distributions of grain sizes for specimens RR 2.8 and RR 21.3. In the bottom part, the distributions are normalized by the mean grain size $D_{m}$ and the maximum likelihood estimate for a log-normal distribution computed thanks to the MATLAB software (R2015a, MathWorks, Natick, Massachusetts, US) being superimposed.

The microstructural characterization was made by EBSD in an FEG-SEM. From each EBSD map, the equivalent diameter $D_{g}$ and the mean orientation of each grain were collected using a post-processing software (Channel 5 (v5.11, Oxford Instrument, High Wycombe, UK) or ATEX [25]). In all the cases, the specimens were fully recrystallized. The specimens with the smallest mean grain size were close to the end of the primary recrystallization. Figure 2 shows examples of obtained grain size distributions for the two samples with the minimum and maximum mean grain size. In the top part of Figure 2, the scales and the bin sizes used to plot the distributions were exactly the same in order to highlight the differences between the obtained microstructures. When normalized by the mean grain size, all the distributions look, however, pretty similar and follow approximately log-normal probability density functions (see the bottom part of Figure 2).

**Figure 3:**
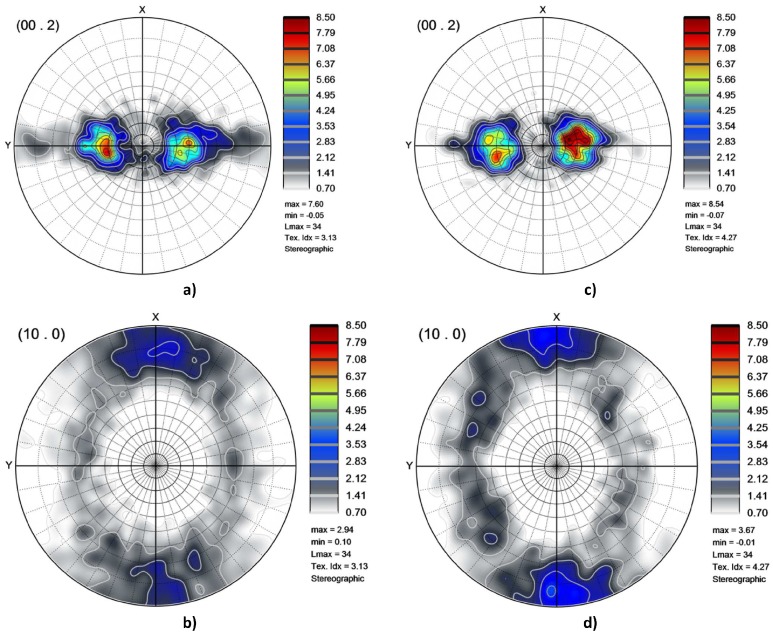
Pole figures for the specimens RR 2.8 (**a**,**b**) and RR 11.7 (**c**,**d**) plotted thanks to the ATEX software [25]. *x* represents the last rolling direction and *y* the transverse direction.

For each specimen, the data set of the grains (volume fractions and orientations) was used to plot the texture. An example is given in Figure 3 in terms of pole figures. The pole figures correspond to well known textures for this material [17,26,27,28,29,30] where basal planes are tilted $30^{\circ }\pm 10^{\circ }$ from the normal toward the transverse direction. When the grains are small, which means that the annealing was stopped close to the end of the primary recrystallization, the texture resembles the deformation one whereas the grain growth texture is obtained after heat treatments made at higher temperatures (see [31] for further information). The data set of the grains (grain diameters in addition to volume fractions and orientations) are the microstructural information used further in the simulations.

### 2.2. Micromechanical Modeling Including Grain Size Effects

The model used to simulate the tensile behavior is a modified version of the one that was developed in [19] to study hardening mechanisms in cp Ti. The purpose of the modification is to account for grain size effects. The model is still based on an advanced elasto-viscoplastic self-consistent (EVPSC) homogenization scheme which considers a small strain setting and an affine linearization of the viscoplastic flow rule. The 1-site self-consistent approximation is formulated thanks to the translated field method [19,23,24]. Compared to hereditary approaches [32], the numerical implementation of such internal variable approach is much easier as no use of Laplace–Carson transform is needed. In the present model, each grain is represented by a sphere to which is associated a mean crystallographic orientation, a volume fraction and a diameter $D_{g}$ which hence allows for considering grain size distribution effects. The EVPSC model has been described in detail in several references [19,23,24], and therefore only the main equations are recalled here.

#### 2.2.1. Micro-Macro Scale Transition

In homogenization theory, the macroscopic stress rate and strain rate tensors, $\dot{\sigma }$ and $\dot{\varepsilon }$, of a representative volume element *V* are obtained by volume averaging the local stress rate and strain rate tensors, $\dot{\sigma_{l}}$ and $\dot{\varepsilon_{l}}$ (scale of the grains), as follows:

(1) $$\dot{\sigma }=\frac{1}{V}\int_{V}{\dot{\sigma }}_{l}\left(x\right)dx,\dot{\varepsilon }=\frac{1}{V}\int_{V}{\dot{\varepsilon }}_{l}\left(x\right)dx.$$

Within the small strain framework, the local total strain rate relative to an elasto-viscoplastic behavior splits into an elastic part and a viscoplastic one:

(2) $$\dot{\varepsilon_{l}}={\dot{\varepsilon }}_{l}^{e}+{\dot{\varepsilon }}_{l}^{p}.$$

The linear Hooke’s law relates ${\dot{\varepsilon }}_{l}^{e}$ to the local Cauchy stress rate ${\dot{\sigma }}_{l}$:

(3) $${\dot{\varepsilon }}_{l}^{e}=s:{\dot{\sigma }}_{l},$$

where the colon : denotes the contracted product of two tensors, and s is the local elastic compliance tensor. The viscoplastic strain rate ${\dot{\varepsilon }}_{l}^{p}$ is a nonlinear tensorial function of the Cauchy stress $\sigma_{l}$. Using a first order affine linearization, it can be written:

(4) $${\dot{\varepsilon }}_{l}^{p}=m:\sigma_{l}+\dot{\eta },$$

where m is the tangent viscoplastic compliance tensor defined by $m=\partial {\dot{\varepsilon }}_{l}^{p}/\partial \sigma_{l}$, and $\dot{\eta }$ corresponds to a back-extrapolated strain rate. In the absence of volume forces, static equilibrium conditions impose:

(5) $$\mathrm{div}\sigma_{l}=0,\mathrm{div}\dot{\sigma_{l}}=0,$$

whereas kinematic compatibility gives:

(6) $$\dot{\varepsilon }=\frac{1}{2}\left(▽\dot{u}+{}^{t}▽\dot{u}\right),$$

where $\dot{u}$ is the material velocity field. For uniaxial tensile tests along the $x_{1}$-axis, mixed boundary conditions are prescribed as follows: ${\dot{\varepsilon }}_{11}=$ experimental applied strain rate and ${\dot{\sigma }}_{22}={\dot{\sigma }}_{33}={\dot{\sigma }}_{23}={\dot{\sigma }}_{31}={\dot{\sigma }}_{12}=0$.

As a result of the translated field method and self-consistent approximations, the explicit expressions of ${\dot{\sigma }}_{l}$ and ${\dot{\varepsilon }}_{l}$ can be obtained in each grain as a function of $\dot{\sigma }$, $\dot{\varepsilon }$, crystallographic orientation and stress history $\sigma_{l}$ in the grain [19,23,24]. These expressions account for coupled elastic and viscoplastic inter-granular accommodations.

#### 2.2.2. Single Crystal Constitutive Laws

Crystal elasticity and plasticity were considered at the single crystal level. Plastic strain and plastic rotation $\omega_{l}^{p}$ can occur by dislocation-based crystallographic slip only:

(7) $${\dot{\varepsilon }}_{l}^{p}=\sum_{s}R^{\left(s\right)}{\dot{\gamma }}^{\left(s\right)},{\dot{\omega }}_{l}^{p}=\sum_{s}S^{\left(s\right)}{\dot{\gamma }}^{(s).}$$

${\dot{\gamma }}^{\left(s\right)}$ denotes the slip rates on systems (s) while $R^{\left(s\right)}$ and $S^{\left(s\right)}$ are, respectively, the symmetric and the skew-symmetric Schmid orientation tensors associated to system (s). Glide on prismatic, basal, pyramidal <a>, 1st order pyramidal <c+a> and 2nd order pyramidal <c+a> slip systems were taken into account. Twinning was not considered since the twin volume fraction in cp Ti was observed to be very small during tensile tests, especially along the rolling direction [17,19]. The slip rates result from the Orowan relation:

(8) $${\dot{\gamma }}^{\left(s\right)}=\rho_{m}^{\left(s\right)}b^{\left(s\right)}v^{\left(s\right)},$$

where $b^{\left(s\right)}$ is the Burgers vector magnitude. From relation 8, it is seen that the evolution of mobile dislocation densities $\rho_{m}^{\left(s\right)}$ and dislocation velocities $v^{\left(s\right)}$ are treated separately on each slip system. This allows for accounting for an initial fast multiplication of mobile dislocations, as suggested by Conrad [33] and Naka [34] to explain the presence of a yield plateau in Ti. When dislocations multiply rapidly, the actual strain rate may overcome the imposed strain rate. As a consequence, the average dislocation velocity has to be adjusted so as to retrieve the imposed strain rate, which can cause a significant slow-down of the flow stress increase. The average dislocation velocity on system (s) was assumed to follow a classic power law relationship:

(9) $$v^{\left(s\right)}=v_{0}^{\left(s\right)}{\left|\frac{\tau^{\left(s\right)}}{\tau_{c}^{\left(s\right)}}\right|}^{n^{\left(s\right)}}\mathrm{sgn}\left(\tau^{\left(s\right)}\right),$$

where $v_{0}^{\left(s\right)}$ is a reference velocity, $n^{\left(s\right)}$ the inverse of the strain rate sensitivity and $\tau^{\left(s\right)}$ the resolved shear stress. It is noteworthy that the components of the tangent viscoplastic compliance tensor m and of the back-extrapolated strain rate tensor $\dot{\eta }$ (Equation (4)) are deduced from the combination of Equations (7)–(9) [19]. Although in elasto-viscoplasticity all non-zero shear stress levels produce some slip, $\tau_{c}^{\left(s\right)}$ can be described as the critical resolved shear stress (CRSS) given the values considered for $n^{\left(s\right)}$ (see further Table 2). It writes as the sum of the lattice friction stress $\tau_{0}^{\left(s\right)}$ and a generalized dislocation strengthening relation that accounts for dislocation interactions between systems [35,36]. Moreover, following references [2,4,10,11,12,13], $\tau_{c}^{\left(s\right)}$ is also assumed to depend on the individual grain size $D_{g}$ in a Hall–Petch type relationship:

(10) $$\tau_{c}^{\left(s\right)}=\tau_{0}^{\left(s\right)}+\mu^{\left(s\right)}b^{\left(s\right)}\sqrt{\sum_{l}a^{\left(sl\right)}\rho_{f}^{\left(l\right)}}+\frac{k_{HP}}{\sqrt{D_{g}}}.$$

$a^{\left(sl\right)}$ denotes the interaction coefficient which is related to the strength of the interaction between system (s) and (l). $\mu^{\left(s\right)}$ is the directional shear modulus of system (s). $\rho_{f}^{\left(l\right)}$ depicts forest (or sessile) dislocation density on system (l). $k_{HP}$ is a Hall–Petch type slope resolved on slip system (s). It is noteworthy that, with this supplementary term, the CRSSs are different from grain to grain according to both their orientation and their size. For the same orientation, the smallest grains are harder than the largest ones, which is in agreement with recent experimental characterizations of CRSSs in grade 1 cp Ti by high energy X-ray diffraction microscopy [37]. Moreover, it must be underlined that, through Equation (10), <c+a> systems will display stronger hardening by virtue of the values of Burgers vector magnitude and directional shear modulus (see Table 2), which is in accordance with experimental observations [38,39]. Finally, both mobile and sessile dislocation densities evolve with plastic deformation [19,40]:

(11) $${\dot{\rho }}_{m}^{\left(s\right)}=\frac{1}{b^{\left(s\right)}}\left[\frac{C_{1}^{\left(s\right)}D_{g}}{b^{\left(s\right)}}-\frac{1}{L^{\left(s\right)}}\right]\left|{\dot{\gamma }}^{\left(s\right)}\right|,$$

(12) $${\dot{\rho }}_{f}^{\left(s\right)}=\frac{1}{b^{\left(s\right)}}\left[\frac{1}{L^{\left(s\right)}}-2k_{c}^{\left(s\right)}b^{\left(s\right)}\rho_{f}^{\left(s\right)}\right]\left|{\dot{\gamma }}^{\left(s\right)}\right|.$$

The negative terms in Equation (11), which correspond to mobile dislocation immobilization, appear as positive terms in Equation (12) and stand for storage of forest dislocations. The mean free path of mobile dislocations $L^{\left(s\right)}$ is assumed to depend on reactions with other mobile dislocations, which are considered through the parameter $C_{2}^{\left(s\right)}$, and interactions with forest dislocations, which are taken into account through the interaction coefficients $a^{\left(sl\right)}$ and the constant $K^{\left(s\right)}$:

(13) $$\frac{1}{L^{\left(s\right)}}=b^{\left(s\right)}C_{2}^{\left(s\right)}\rho_{m}^{\left(s\right)}+\frac{\sqrt{\sum_{l}a^{\left(sl\right)}\rho_{f}^{\left(l\right)}}}{K^{\left(s\right)}}.$$

$k_{c}^{\left(s\right)}b^{\left(s\right)}$ represents the annihilation distance between dislocations and the term associated to it thus accounts for dynamic recovery.

In Equation (11), the mobile dislocation density production is related to the term $C_{1}^{\left(s\right)}D_{g}$ and thus is set proportional to the grain diameter. The idea underlying this formalism is that the expansion of dislocation loops (e.g., from a Frank–Read source) is less constrained in larger grains. In smaller grains, dislocation loops will pile-up (as observed in cp Ti [41]) or will be absorbed [42,43,44] at the grain boundary sooner, i.e., for a smaller increase of dislocation length (= smaller increase of dislocation density). The mechanism of loops expansion should be preserved in cp Ti down to grains of 1 µm or lower (the obstacles spacing *d* for line tension strengthening may be estimated as $d=2\mu^{\left(s\right)}b^{\left(s\right)}/\tau^{\left(s\right)}$, which gives d≈ 0.3 µm for prismatic systems with $\tau^{\left(s\right)}=150$ MPa). Furthermore, with decreasing grain size, the ratio of grain boundary area over grain volume increases. Hence, the probability of dislocation absorption by grain boundaries should increase when the grain size decreases. Through Equations (11)–(13), it is observed that the grain size, by influencing the increase rate of mobile dislocation densities, will also affect the evolution of forest dislocation densities and hence the hardening behavior. Finally, one may argue that the mean free path of dislocations (Equation (13)), in addition to being dependent on dislocation density, should also depend on the grain size. Accordingly, a term that scales as $1/D_{g}$ could have been added in Equation (13) as well, as was done for instance in [4]. However, by doing so, one would get higher forest dislocation densities, and thus stronger work hardening in small grain samples, which is the opposite of what is observed (see Figure 1 and further). Hence, our modelling assumption is that the main effect of grain boundaries in cp Ti is to act as barriers or sinks for dislocations [41,42,43,44] which, as a consequence, restricts the expansion of dislocation length, rather than having a significant contribution to the creation of forest dislocations, which is mainly accounted for by dislocations’ interactions.

#### 2.2.3. Model Parameters

The model is coded in Fortran and solved in an explicit way using very small time steps. It was checked that convergences of the solutions were indeed reached. The values of the model’s parameters are given in Table 2 (parameters specific to slip families) and Table 3 (parameters not specific to slip families). Except for $\tau_{0}^{\left(s\right)}$, $\rho_{f_{0}}$, $C_{1}$ and $k_{c}$, these values are identical as in [19]. It was shown in [19] that the above described constitutive setting (without the incorporation of grain size effects) was able to explain the opposite effect of strain rate with regard to the orientation of the tensile axis on the three-stage hardening behavior of cp Ti thanks to the consideration of lower strain-rate sensitivity for prismatic systems. Because of the presence of the Hall–Petch type term in the expression of $\tau_{c}^{\left(s\right)}$ (Equation (10), the values of $\tau_{0}^{\left(s\right)}$ and $\rho_{f_{0}}$ had to be modified to retrieve CRSSs with about the same level as in [19]. The value of the resolved Hall–Petch type slope $k_{HP}$ corresponds approximately to the macroscopic Hall–Petch slope determined in [31] divided by the Taylor factor. The value of $C_{1}$ was adjusted so that the product $C_{1}D_{g}$ is about the former value of $C_{1}$ in [19] for a mean grain size of 10 µm.

## 3. Results and Discussion

**Figure 4:**
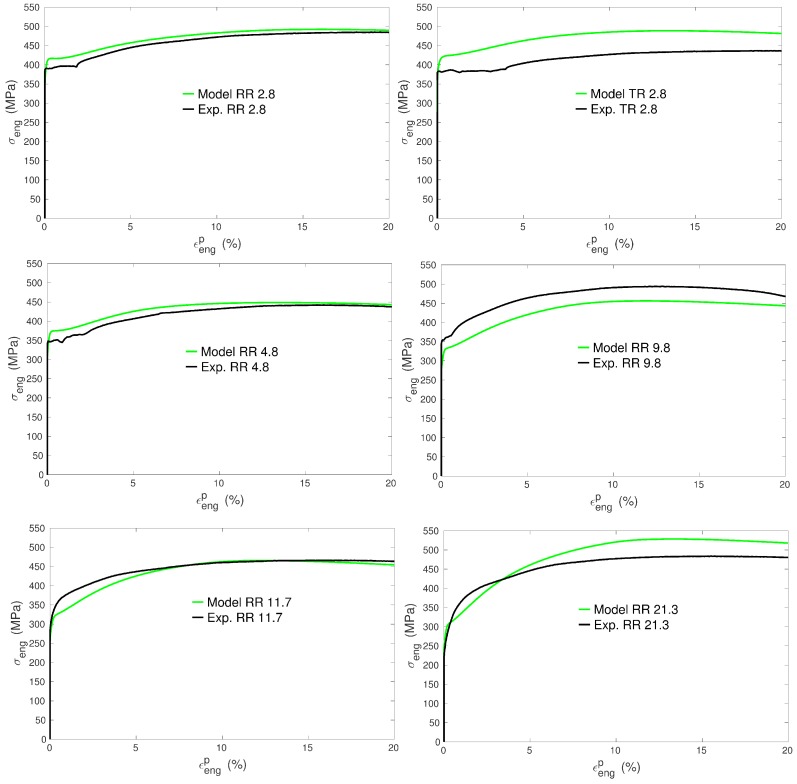
Tensile curves (engineering stress and strain) for model predictions and experimental measurements for the six specimens deformed along the last rolling direction.

**Figure 5:**
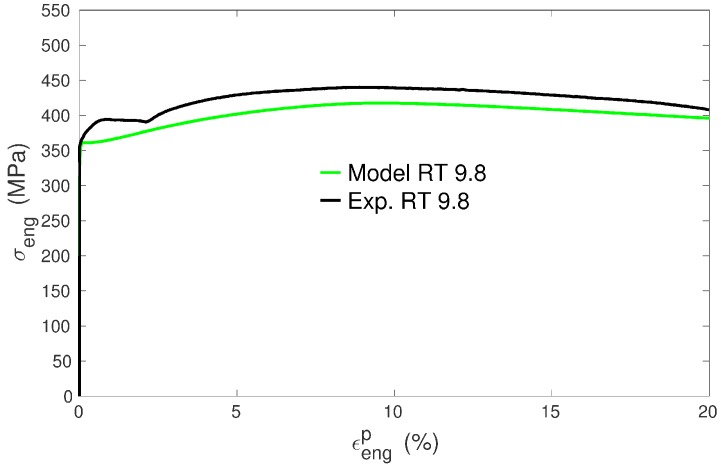
Tensile curves (engineering stress and strain) for model predictions and experimental measurements for the specimen deformed along the last transverse direction.

The tensile curves of the seven specimens were simulated using the same set of model parameters. The specific set of grain orientations, volume fractions and equivalent diameters was used for each specimen. The numbers of grains considered in the simulations were the numbers of grains of the EBSD maps indicated in Table 1. Figure 4 and Figure 5 show the simulated curves and their equivalent experimental counterparts. In order to characterize further the mechanical behavior, several quantities were collected from the tensile curves:

- the 0.2% yield stress: $\sigma_{e}$,
- the maximal engineering stress: $\sigma_{max}$,
- the $\sigma_{max}$ corresponding engineering strain: $\varepsilon^{*}$,
- the hardening amplitude: $\Delta \sigma =\sigma_{max}-\sigma_{e}$.

**Figure 6:**
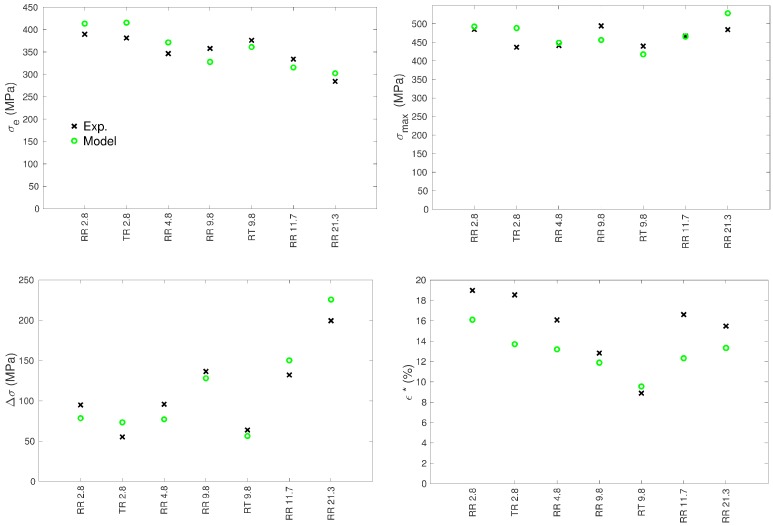
Comparisons between experimental and calculated values of $\sigma_{e}$, $\sigma_{max}$, Δσ and $\varepsilon^{*}$ (see text for definitions).

Both the experimental and simulated values of these quantities are reported in Figure 6 for the seven specimen.

### 3.1. Yield Stress

**Figure 7:**
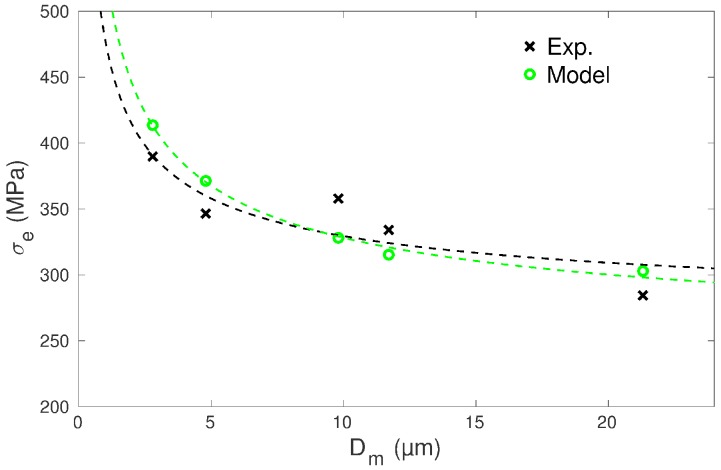
Comparisons of experimental and calculated values of the yield stress $\sigma_{e}$ with respect to the mean grain size $D_{m}$ for the specimens of RR type. Linear fit estimates obtained by MATLAB between $\sigma_{e}$ and $1/\sqrt{D_{m}}$ are shown in green (Model) and black (Exp.) dotted lines.

The experimental yield stress increases with decreasing mean grain size $D_{m}$ and follows rather well a Hall–Petch relationship (Figure 7), i.e., a scaling $\sigma_{e}∼1/\sqrt{D_{m}}$, as it was already shown on a larger data set of cp Ti specimens that also included partially recrystallized microstructures [31]. The calculated yield stresses follow a pretty correct trend considering the unlikely ranking of measured yield stresses between samples RR 4.8 and RR 9.8 (Figure 6 and Figure 7). The assumption made for the dependence of the CRSS on the grain size (Equation (10)) thus appears relevant. As also found in earlier works [2,4,10,11,12,13], it is confirmed that the consideration of a Hall–Petch type dependence at the scale of slip systems enables for retrieving a Hall–Petch law at the macroscopic scale (Figure 7). This transition may, however, not be as direct as it seems since the macroscopic Hall–Petch law deals with the mean grain size, whereas the individual grain size is considered at the scale of slip systems. Hence, the smallest grains of the distribution might act as limiting factors for the complete diffusion of plasticity in the sample.

As already observed [17,19,45], for the same mean grain size, the yield stress is higher when the tensile axis is parallel to the transverse direction (comparisons of samples RT 9.8 and RR 9.8) due to less-well oriented easy prismatic systems.

### 3.2. Local Mechanical Fields

**Figure 8:**
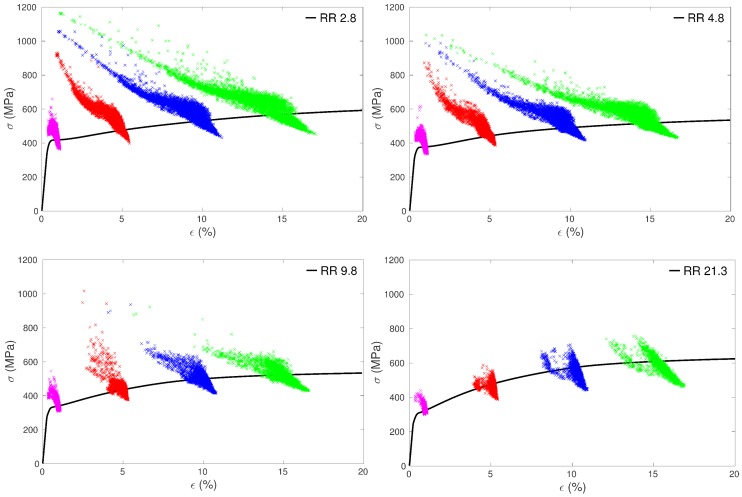
Distribution of predicted tensile stress–strain relationships for each grain at 1% (magenta), 5% (red), 10% (blue) and 15% (green) of macroscopic strain for specimens RR 2.8, RR 4.8, RR 9.8 and RR 21.3. The corresponding model tensile curve is superimposed on each plot (black thick line).

Figure 8 shows the evolution of the tensile component of local stresses and local strains in the grains for specimens with different average grain sizes. Each dot represents the local stress and local strain values in a given grain. As can be seen, the clouds formed by these dots become larger and larger with increasing macroscopic strain, while their shapes evolve from vertically elongated towards more and more horizontally spread. This result is radically different from the predictions of a simple Taylor model, which would give dots aligned along vertical lines at any strain (uniform strain). The results shown in Figure 8 are in agreement with strain measurements obtained locally in polycrystalline materials [46,47,48], especially for hexagonal structure [49]. The stress and strain fluctuations arise in the present modeling because of the consideration of non-uniform distributions of grain orientations and sizes coupled with elastic and plastic anisotropies at the single crystal level, as well as grain size dependent CRSSs (Equation (10)) and grain size dependent dislocation density evolution laws (Equation (11)). At 1% macroscopic strain, the fluctuations are mostly due to elastic incompatibilities. With increasing deformation, plastic strains become rapidly larger than elastic strains and plastic incompatibilities become predominant. Moreover, as noticed in [2,4], the grain size dispersion strongly influences the local mechanical fields. Actually, for a same grain size dispersion, $\left(D_{max}-D_{min}\right)/D_{m}$, the relative dispersion of the Hall–Petch type term, $k_{HP}/\sqrt{D_{g}}$ in Equation (10), is invariant whatever the mean grain size is. Since the contribution of the Hall–Petch type term increases with decreasing grain size, the absolute dispersion of the CRSSs becomes larger and larger in samples having smaller and smaller mean grain sizes (considering that the normalized grain size distributions of our samples are quite similar (Figure 2)). It was checked that this effect alone cannot explain totally the impressive increase in the scattering of the local strains and stresses when decreasing the mean grain size in Figure 8. Indeed, there is an additional effect that is related to the grain size dependence of the mobile dislocation density evolution (Equation (11)). The latter impedes significantly the multiplication of mobile dislocations in the smallest grains (see further Figure 11) and thus restricts the value of the slip rates in those grains (Equation (8)). For instance, the smallest grain diameter considered in our simulations, 0.45 µm in sample RR 2.8, is relatively close to the value of 0.14 µm below which ${\dot{\rho }}_{m}^{\left(s\right)}$ is initially negative in Equation (11). As a result, local stresses almost as high as 1200 MPa are reached in some grains of sample RR 2.8 at a macroscopic strain of 15% (Figure 8). These grains are very small and act like hard particles as they exhibit very little deformation (the corresponding local strains are barely greater than 1%). They represent, however, a very small volume fraction of the whole sample.

### 3.3. Slip Activity

**Figure 9:**
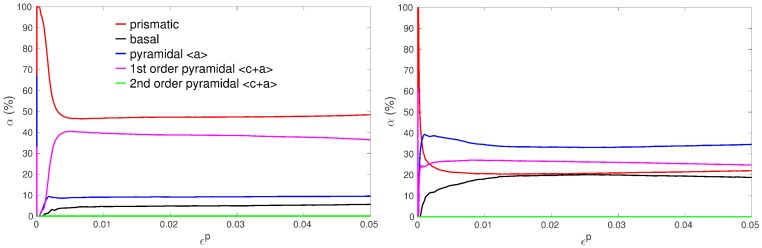
Relative activities of slip families (α) predicted by the model for specimen RR 9.8 (**left**) and RT 9.8 (**right**).

From the simulations, it is possible to estimate the relative activity α of a slip family as:

(14) $$\alpha =\frac{\sum_{g=1}^{ng}\sum_{s=p}^{q}f_{g}\left|{\dot{\gamma }}_{g}^{\left(s\right)}\right|}{\sum_{g=1}^{ng}\sum_{s=1}^{ns}f_{g}\left|{\dot{\gamma }}_{g}^{\left(s\right)}\right|},$$

where the slip system numbers of a specific family goes from *p* to *q*. ns is the total number of slip systems, ng the total number of grains and $f_{g}$ the grain volume fraction. The distribution of the slip activity is mainly influenced by the texture, the initial values of the CRSSs and the intergranular stresses that arise from strain incompatibilities. For tensile tests along the rolling direction, it is known that, as a result of the texture, a vast majority of the grains have large macroscopic Schmid factors for prismatic, pyramidal <a> and 1st order pyramidal <c+a> glide and low ones for basal slip [17,31]. On the contrary, along the transverse direction, there is no big difference of Schmid factors among the four main slip families, 1st order pyramidal <a> and <c+a> being nevertheless the most favorably oriented ones [17,31]. As a consequence, for deformation simulation along the last rolling direction, prismatic slip strongly dominates at the very beginning of deformation because of its low CRSS value. Then, it decreases to reach a quasi steady-state of about 50% of the total slip activity whatever the mean grain size (Figure 9). The second most important contribution to plastic deformation arises from 1st order pyramidal <c+a> systems, which exhibit a maximum around 0.5% plastic strain with a rapid initial increase before and a very slight steadily decrease after. This decrease is probably due to the stronger hardening of <c+a> systems. Pyramidal <a> and basal systems have also non negligible contributions, while the one of 2nd order pyramidal systems is completely unimportant due to the much higher value of its CRSS. During the simulations, dislocations of 2nd order pyramidal systems act thus only as a static forest. For deformation along the transverse direction, the fall of the prismatic activity is much stronger and occurs sooner. In steady-state, the contribution of the four main slip families are between 20% and 35%.

### 3.4. Hardening Behavior

**Figure 10:**
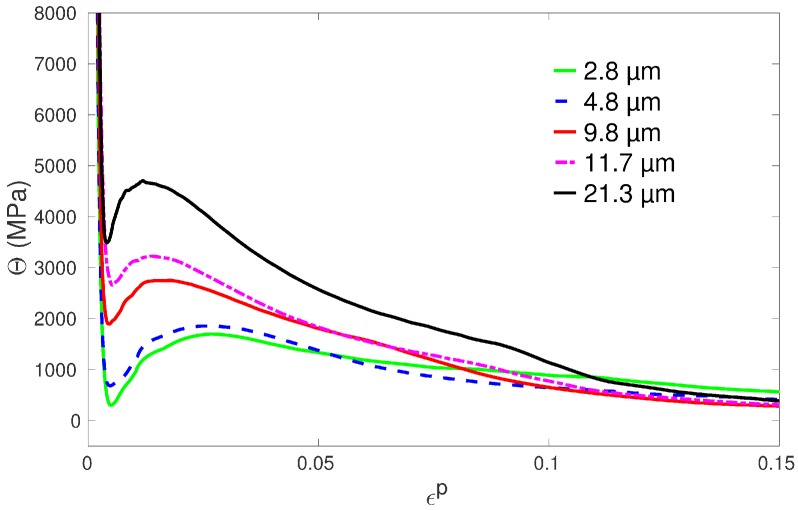
Model predictions for the work hardening rate evolution for the specimens of RR type.

**Figure 11:**
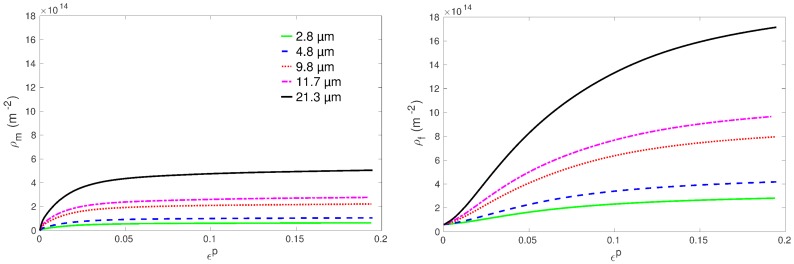
Model predictions for the evolution of the total forest ($\rho_{f}$) and mobile ($\rho_{m}$) dislocation densities for the specimens of RR type. Densities are cumulated over the 30 slip systems and averaged over the grains’ population.

It can be noticed from the tensile experimental curves (Figure 1 and Figure 4) that the tendency to form an initial plateau becomes more and more pronounced with decreasing mean grain size. This general trend is in agreement with other experiments in titanium [50] and was also characterized as such in copper [9] for instance. The model reproduces very well this trend (Figure 4). Moreover, the evolution of the work hardening rate $\theta =d\sigma /d\varepsilon^{P}$ for all the simulations of RR type is plotted in Figure 10. In agreement with the present experiments and previous tensile tests on cp Ti [17,19,29,30], a three-stage work hardening behavior is obtained, where an initial fall of θ is followed by an increase and a final progressive decrease. More importantly, the model predicts a growing well depth with decreasing grain size, or, in other words, a transient low work hardening rate with smaller and smaller value as the grain size decreases. In Figure 10, it is also noteworthy that the predicted work hardening rates of all the specimens converge at high strains, meaning that the model correctly predicts an insensitivity to grain size at high strains [9]. The reason is that the mobile dislocation density reaches a saturation value (see further Figure 11).

The low point of the work hardening rate precisely corresponds to the moment when the activity of the 1st order pyramidal <c+a> systems approaches its maximum value, around 0.5% of plastic strain (Figure 9). Hence, the appearance of a low point in the work hardening rate evolution seems to be related to the start of multiple slip within grains, or to a kind of balance between <a> and <c+a> slip activities. Under such conditions, plastic strain incompatibilities and the induced intergranular stresses are strongly decreased. Along with the fast multiplication of mobile dislocations (Equation (11)), this gives rise to an initial state with very weak hardening. In agreement with previous experiments, when the tensile axis is parallel to the transverse direction (comparisons of samples RT 9.8 and RR 9.8), the plateau effect is even more pronounced due to an even more balanced slip activity distribution (Figure 9). This texture effect is very well reproduced by the model (Figure 5).

The grain size effect on the work hardening rate can be understood by analyzing the predicted evolutions of dislocation densities in Figure 11. As expected from Equations (11) and (12), the assumption of a mobile dislocation density production that grows with grain size makes both mobile and forest dislocation densities increase with the grain size. This implies that the rate of CRSS increase is more important in large grains since $\tau_{0}^{\left(s\right)}$ and the Hall–Petch type term do not evolve with deformation (Equation (10)). The increase of CRSSs counterbalances the previously mentioned effects, which lead to a low point in the work hardening rate evolution. As a result, the model indeed predicts a less and less pronounced transient low work hardening rate as the grain size increases.

The same explanation holds to interpret the effect of the grain size on the hardening capacity. Both the observations and the model predictions indicate a hardening amplitude Δσ that tends to increase with the grain size (Figure 6). The CRSSs of the small grains are initially higher than those of the large grains while the latter can display larger increases. By consequence, large grains exhibit a higher hardening capacity.

In addition, the correct prediction of a smaller hardening amplitude obtained for sample RT 9.8 (Figure 5 and Figure 6) results from the choice of a greater $C_{1}$ value for prismatic systems compared to other slip families (Table 2). Indeed, since the contribution of prismatic slip is less important for deformation along the transverse direction (Figure 9), this assumption leads to much lower dislocation densities in sample RT 9.8 compared to RR 9.8 and thus to much weaker work hardening (see [19] for further information).

Finally, it can be observed that the model reproduces the Considere point ($\sigma_{max}$, $\varepsilon^{*}$), i.e., a limit for the homogeneous deformation, which matches satisfactorily the experimental results. The maximal engineering stress, $\sigma_{max}$, compares rather well with experiments, whereas the corresponding engineering strain, $\varepsilon^{*}$, is slightly underestimated except for the case RT 9.8 (extension along the transverse direction) (Figure 6). The model, with its assumptions and with the used parameters, is thus able to account for the balance of appearing and disappearing dislocations. The value of $\varepsilon^{*}$ is mainly controlled by the dynamic recovery parameter $k_{c}$ (Equation (12)). $k_{c}$ is set to one unique value all along the deformation, which obviously does not allow for capturing totally the complexity of the dynamics of the recovery process (e.g., effect of thermally-activated cross-slip).

## 4. Conclusions

This study considers tensile curves of a set of cp Ti specimens with different microstructures, especially with several mean grain sizes. It is observed that the yield stress depends on the grain size following a Hall–Petch relationship. The hardening behavior shows also some dependence with the grain size. The tendency of stress–strain curves to form a plateau becomes more and more pronounced with decreasing mean grain size while the hardening amplitude $\Delta \sigma =\sigma_{max}-\sigma_{e}$ increases with the grain size. All these observations are well reproduced by an EVPSC model that incorporates grain size effects in a crystal plasticity framework where dislocation densities are the state variables. While the CRSSs are made dependent on the individual grain size through the addition of a Hall–Petch type term (Equation (10)), the originality of the model comes from the fact that the multiplication of mobile dislocation densities is also made grain size dependent (Equation (11)). Our assumption is that, due to grain boundaries acting mainly as barriers or sinks for dislocations, the smaller the grain size, the smaller the expansion of dislocation loops and thus the smaller the increase rate of mobile dislocation density. As a consequence of this assumption, both mobile and forest dislocation densities increase with the grain size, which provides an explanation for the grain size dependence of the transient low work hardening rate and hardening amplitude. It is noteworthy, however, that, in agreement with observations, the predicted work hardening rates of different mean grain size samples eventually converge at high strains because the mobile dislocation density reaches a saturation value. The fact that the model provides correct estimates of the homogeneous deformations is also quite positive and shows that the formalism, as well as the choice of the parameters, reflects rather well the complex reality.

## Figures and Tables

**Table 1:** Preparation conditions and some metallurgical data of the seven specimens.

Specimen	Cold Rolling Reduction	Annealing Conditions	Number of Grains in the Data Set
RR 2.8	75%	500 °C - 40 mn	6254
TR 2.8	75%	500 °C - 40 mn	7328
RR 4.8	75%	650 °C - 1 h	8793
RR 9.8	75%	730 °C - 2 h	3262
RT 9.8	75%	730 °C - 2 h	3262
RR 11.7	75%	740 °C - 2 h	4075
RR 21.3	30%	840 °C - 4 h	1273

**Table 2:** Model parameters that are specific to slip families (Prismatic: *P*, Basal: *B*, Pyramidal <a>: $\Pi_{1}^{<a>}$, 1st order Pyramidal <c+a>: $\Pi_{1}^{<c+a>}$, 2nd order Pyramidal <c+a>: $\Pi_{2}^{<c+a>}$).

	*P*	$\Pi_{1}^{<a>}$	*B*	$\Pi_{1}^{<c+a>}$	$\Pi_{2}^{<c+a>}$
*b* (Å)	2.95	2.95	2.95	5.53	5.53
μ (GPa)	35.0	37.1	46.5	47.7	49.2
$\tau_{0}$ (MPa)	50	90	120	75	150
$C_{1}$ ($m^{-1}$)	80	15	15	15	15
*n*	65	32	32	32	32

**Table 3:** Model parameters that are not specific to slip families. $C_{ij}$ are the stiffness constants. $a_{coli}$ denotes the interaction coefficient related to collinear interactions, i.e., reactions between dislocations with parallel Burgers vectors gliding in different planes, whereas $a_{\neq coli}$ refers to non-collinear interactions (see details in [19]).

c/a	$C_{11}$ (GPa)	$C_{33}$ (GPa)	$C_{44}$ (GPa)	$C_{12}$ (GPa)	$C_{13}$ (GPa)	$k_{HP}$ (MPa.m^0.5^)	
1.587	160	181	46.5	90	66	0.1	
$v_{0}$ (ms^−1^)	$\rho_{m_{0}}$ (m^−2^)	$\rho_{f_{0}}$ (m^−2^)	$C_{2}$	K	$a_{\mathrm{coli}}$	$a_{\neq \mathrm{coli}}$	$k_{c}$
$3\times 10^{-5}$	$1\times 10^{10}$	$2\times 10^{12}$	75	80	0.7	0.1	10

## Abbreviations

The following abbreviations are used in this manuscript:

Ti	titanium
cp	commercially pure
RD	rolling direction
TD	transverse direction
EBSD	electron backscatter diffraction
SEM	scanning electron microscopy
FEG	field emission gun
EVPSC	elasto-visco-plastic self-consistent
CRSS	critical resolved shear stress
